# New York Homœopathic Union

**Published:** 1892-06

**Authors:** L. M. Stanton

**Affiliations:** Sec’y.; 181 West 87th Street, New York


### NEW YORK HOMŒOPATHIC UNION.

The regular monthly meeting of the New York Homœopathic Union was held at the office of Dr. Edmund Carleton, 53 West Forty-fifth Street, New York, April 21st, 1892. Present: Dr. B. Fincke in the Chair; Drs. Baylies, Carleton, Clark, Dyer, Morgan, O'Brien, O'Connor; also the following visitors: Dr. Cartier, of Paris, who is here to observe the workings of pure Homœopathy; Dr. Kortwright, of Susquehanna, Pa.; Dr. Santee, of Cortland, N. Y.

The Secretary, Dr. Stanton, was unable to attend the meeting. Dr. Carleton was appointed temporary Secretary, and took the following minutes.

The President announced the fourth anniversary of our Union, and that the first business would be the election of officers for the ensuing year. Postponed to the next meeting.

The President said that great interest had lately been revived among German homœopathic physicians, in Professor Jaeger's experiments with high potencies by neural analysis, to which he has been invited by the publisher of the Leipzig *Allg. Hom. Zeitung*, and of which the first installment has appeared in that journal. Jaeger has now used a pocket chronoscope of his own invention, which works easier than the old Kipp chronoscope ten years ago. At that time he found the greatest action of high potencies of Natrum-mur. in a series of potencies from 30 to 4,000, between 4,000 and 5,000. This caused him, before the meeting of the German physicians and naturalists to declare that the limit of divisibility of matter in consequence of this experiment had to be extended to at least the 4,000th centesimal potency. His present experiments have been instituted with a view to find for the various potencies the point of their indifference, or the point where they cease to act. Professor Jaeger declares emphatically that his experiments enable him to say with certainty, that high potencies possess more and more varified medicinal action than low potencies and crude drugs. This statemeut coming from one of the foremost scientific men of Europe, a pioneer in physiology, and a fearless investigator, is to be hailed as the beginning of a new era of the homœopathic development in Europe, and especially in Germany, where high potencies have been silenced and depressed in every way, till lately they have again come into notice in consequence of the Koch fiasco. The homœopathic physicians, on account of supposed alliance of Isopathy with Homœopathy, taking up this subject, declared in accordance with the allopathic experience of the mortality of the crude tuberculin, that high potencies—1,000 and higher—proved curative; so that the ludicrous aspect is presented of Dr. Koch helping our high potencies to their rightful recognition.

Dr. W. P. Wesselhœft's report to the I. H. A., on the condition of Homœopathy in Germany, has been published in the *Berliner Zeitschrift*, and created a sensation, with, to be hoped for, good results.

*The Organon* was then read and discussed; sections 146 and 153 inclusive, the last not fully, and it was voted to begin with that section at the next meeting.

The words "in the place of" (sec. 148) were carefully considered. Finally all agreed that we cannot understand just how the substitution of remedy for disease is made, though there is no doubt of the substitution.

Dr. Morgan read aloud from Stratton's Translation, section 149 and foot-note. The President, with the German edition and his own literal translation before him, informed the company that in the original Hahnemann had added at the close of the note, the words omitted by Stratton: "The just reward may await them, that when getting sick they may themselves be treated in the same manner."

All present were surprised at the omission, and laughed heartily.

An explanation was asked of the meaning of section 151. Dr. Carleton illustrated by a case from practice. A hard-headed old Scotchman had said to his boon companions last Christmas that he should not drink another drop of whiskey after New Year's day. "Why, Dave!" they exclaimed, "you have drank whiskey for forty years. If you stop now it will kill you." "Very well," was the reply, "then I will die; but I shall not drink any more whiskey." And he stopped drinking.

He became very ill with indigestion, insomnia, and nervousness, and applied to the Doctor for relief. He gave Nux-v. after making comparisons, but without avail. Next Pulsatilla, after careful search, but with slight palliation. That nettled the Doctor, who took the case anew, with great care, and gave the apparent simillimum, Argent-nit. This produced only slight palliation. It was apparent that some important part of the case had been omitted. Accidentally, the patient admitted that his distress was ameliorated by bending double and pressing hard upon the abdomen. He seemed half ashamed and sorry to make the admission, but the grand characteristic had been found. All the symptoms fell into line for *Colocynth*, which cured speedily.

This illustration was considered good.

A general discussion of the value of diagnosis ensued. It was agreed that the diagnosis of a case was valuable, as enabling the physician to avoid laying stress upon the diagnostic symptoms when selecting the remedy; and also as enabling the physician to make prognosis. Dr. Morgan made this very apparent.

The importance of not changing the direction of a disease was brought out. Dr. Dyer illustrated from the practice of the late Dr. Bayard, who was always very particular on that point. He (Dr. B.) had prescribed carefully and successfully for a case of pruritus. Less troublesome symptoms appeared in remote and altogether superficial parts. Instead of letting alone until the cure should be complete, another physician then prescribed for the new symptoms, and so successfully, that they all disappeared; the pruritus returned and could not be cured.

Adjourned.

L. M. STANTON, *Sec'y.*

181 WEST 87TH STREET, NEW YORK.

